# Laparoscopic hydrocelectomy of the canal of Nuck in adult female: Case report and literature review

**DOI:** 10.1016/j.ijscr.2019.11.040

**Published:** 2019-11-27

**Authors:** Fakhar Shahid, Walid El Ansari, Mohamed Ben-Gashir, Abdelrahman Abdelaal

**Affiliations:** a Department of General Surgery, Hamad Medical Corporation, Doha, Qatar; b Department of Surgery, Hamad General Hospital, Hamad Medical Corporation, Doha, Qatar; c College of Medicine, Qatar University, Doha, Qatar; d School of Health and Education, University of Skövde, Skövde, Sweden; e Department of Laboratory Medicine and Pathology, Hamad Medical Corporation, Doha, Qatar

**Keywords:** Hydrocele, Inguinal swelling, Laparoscopic TAPP, Hernia, Adult female, Case report

## Abstract

**Introduction:**

Among adult females, Hydrocele of Canal of Nuck (HCN) is a very rare condition. The majority of the reported cases of HCN were not conclusively diagnosed until surgery was performed on a suspected inguinal hernia. To the best of our knowledge, this is the first case of laparoscopically operated HCN in adult female in Middle East and North Africa Region, and the fourth such case worldwide.

**Presentation of case:**

A 36-year-old female presented with a painful small swelling in the right groin of 3 months duration. The swelling extended to the right labia majora while standing, and disappeared when the patient was in prone position. There was no lymph node enlargement, no other masses in the abdomen, and no swelling in the contralateral side. Ultrasonography was undertaken and the patient was admitted electively for laparoscopic exploration and repair of non-complicated right inguinal hernia. She was known to have dyslipidemia on medications. Further history and physical examination were unremarkable. Intraoperatively it was diagnosed to be right-sided HCN which was excised and repaired by mesh fixation using transabdominal preperitoneal (TAPP) approach. Her follow up course was unremarkable.

**Conclusions:**

This case expands the clinical and intraoperative potential differential diagnosis of adult female groin masses. Surgeons should consider such cases when they observe such swellings intraoperatively. Laparoscopic TAPP approach in such cases is a superior diagnostic and treatment modality.

## Highlights

• A very rare case of hydrocele of canal of Nuck in elderly lady.
• This case expands the clinical and intraoperative potential differential diagnosis of adult female groin masses.
• Hydrocele of canal of Nuck must be considered one of the differential diagnosis in ladies with inguinal masses.
• Laparoscopic TAPP is superior to open approach in managing such cases.
• Probably the first case of hydrocele of canal of Nuck in adult female operated laparoscopically in the Middle East and North Africa (MENA) Region, and the fourth such case worldwide.

## 1 Introduction

In adult females, hydrocele of Canal of Nuck (HCN) is a very rare condition and results from failure of obliteration of the distal portion of the canal, which forms a fluid-containing sac [1]. HCN is often misdiagnosed as an incarcerated inguinal hernia followed by emergency surgery [2]. Most HCN are diagnosed intraoperatively due to very low clinical suspicion [3]. Sometimes Ultrasound is done to narrow down the differential diagnosis [4]. The standard treatment is complete excision of the hydrocele by open surgery, however recently, cases of laparoscopic excision by the TAPP (transabdominal preperitoneal) and TEP (totally extraperitoneal) approaches have also been reported [5]. This paper aims to assist the differential diagnosis and to contribute to the literature by presenting a very rare case of HCN in an adult female, which was laparoscopically excised and the inguinal canal repaired by TAPP in our academic hospital. To the best of our knowledge, this is the first case of HCN in adult female operated laparoscopically in the Middle East and North Africa (MENA) Region, and the fourth such case worldwide. This report also reviewed the published literature to assess the clinical characteristics, presentation, diagnosis and management of laparoscopically removed HCN in adult female. We report this case in line with the updated consensus-based surgical case report (SCARE) guidelines [6].

## 2 Case presentation

A 36 year old female presented to our outpatient surgical clinic at Hamad Medical Corporation in Qatar, complaining of a painful small swelling in the right groin of 3 months duration. The swelling extended to the right labia majora while standing, and disappeared when the patient was in prone position. Past social, family, environmental, trauma and employment histories were unremarkable. She had no history of previous surgeries. Patient was a nonsmoker and had never consumed alcohol. Her past medical history indicated that she had hyperlipidemia, and was under medication, otherwise the patient was not on any other medication.

## 3 Physical examination

Palpation revealed a cystic sausage-shaped swelling (≈6 × 3 cm) in the right groin that was more appreciated while standing and was expansile with cough. The skin overlying the swelling showed no redness. The swelling was reducible and minimally tender on touch. There was no lymph node enlargement, no other masses in the abdomen, and no swelling in the contralateral side. The rest of the physical and neurological examinations were all unremarkable. On admission, her pulse, blood pressure and temperature were normal.

## 4 Investigations

**Fig. 1:**
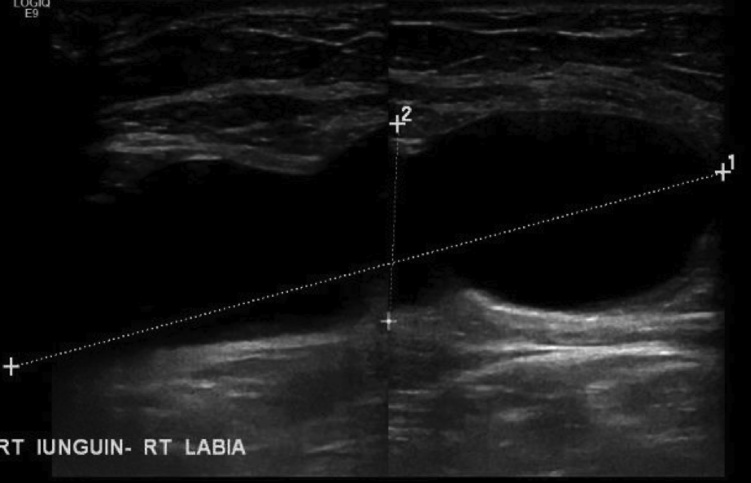
Ultrasound abdomen and pelvis showing right inguino-labial hydrocele.

Her laboratory blood test was in the normal range, except for the lipid profile that showed hyperlipidemia (total cholesterol 9.04 mmol/L, LDL 6.6 mmol/L, normal triglycerides). Ultrasound abdomen and pelvis revealed a cystic structure measuring 8.4 × 2.6 cm extending from right inguinal region to the right labia consistent with right inguino-labial hydrocele (Fig. 1).

## 5 Surgical technique

**Fig. 2:**
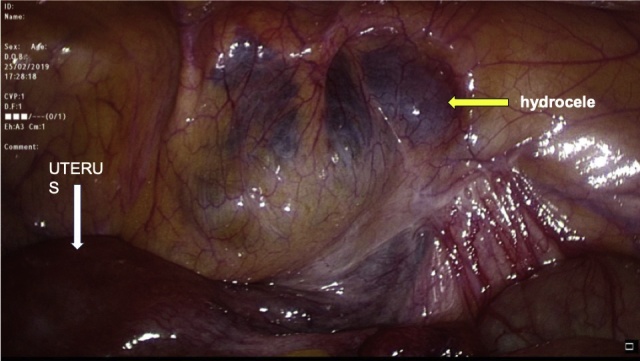
Laparascopic view of the hydrocele (yellow arrow) and uterus (white arrow).

**Fig. 3:**
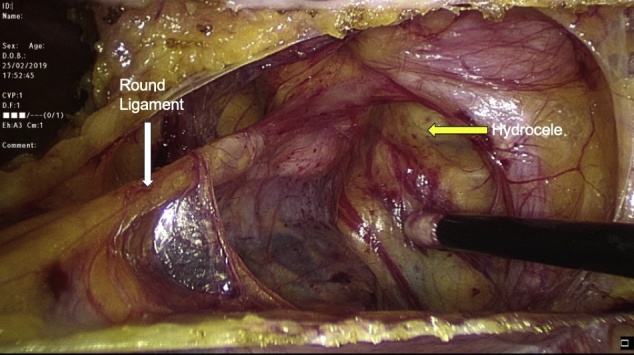
Dissection of the sac (yellow arrow) around the round ligament (white arrow).

**Fig. 4:**
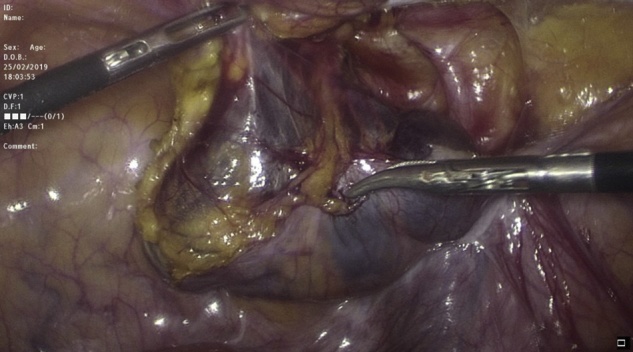
Separation of the sac from the peritoneum covering.

**Fig. 5:**
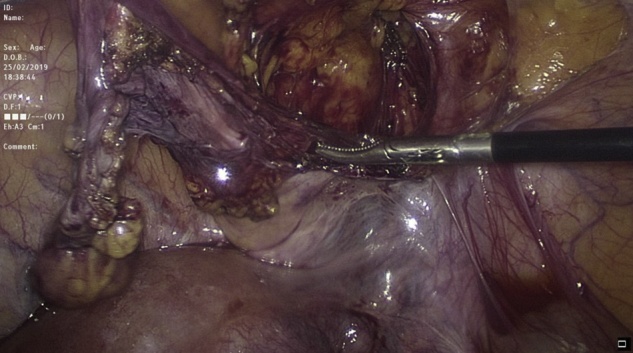
Excision of the sac.

**Fig. 6:**
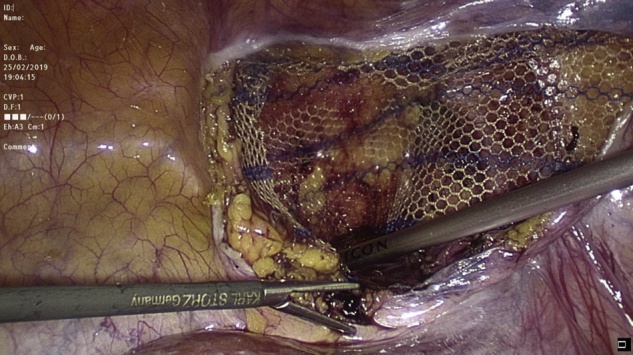
Fixation of mesh to strengthen the posterior inguinal wall.

**Fig. 7:**
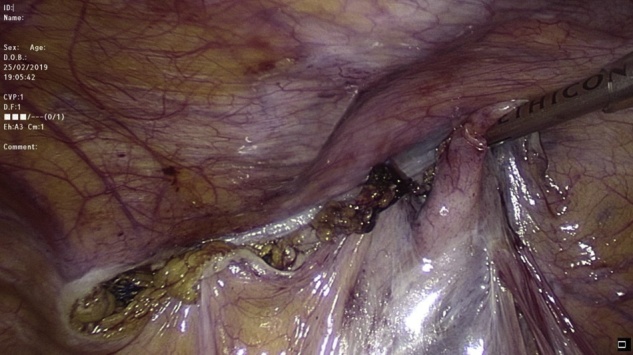
Closure of the peritoneum to cover the mesh.

**Fig. 8:**
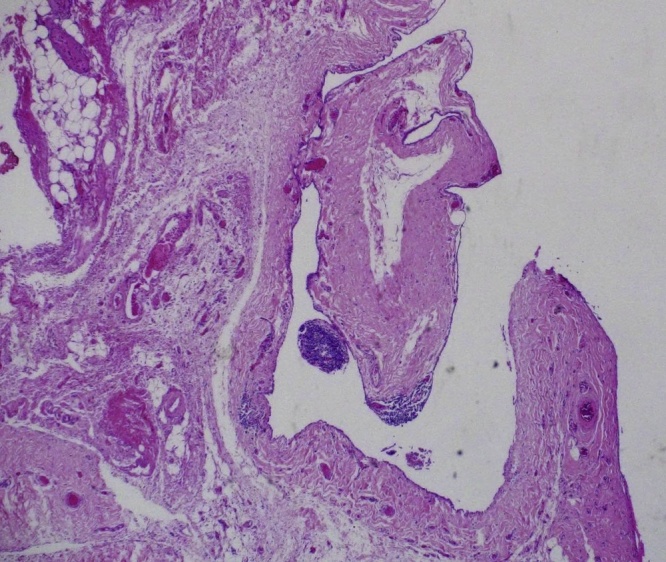
Histopathology showing characteristics consistent with hernia sac and no evidence of granuloma or malignancy.

Transabdominal laparoscopic exploration for excision of the HCN and mesh repair (TAPP repair) was thoroughly discussed with the patient, after which the patient signed the informed consent. The surgery undertaken by an experienced consultant surgeon revealed a retroperitoneal bulge of cystic mass at the right inguinal area lateral to the right inferior epigastric vessels (Fig. 2). Peritoneum was opened by incision from level of anterior superior iliac supine to the medial umbilical ligament. The sac was dissected around the round ligament from the posterior wall of the inguinal canal (Fig. 3, Fig. 4). Then the round ligament was cut and dissection of the sac progressed to completely excise the sac including the round ligament from the labia majora (Fig. 5). Mesh repair using ultrapro mesh of 15 × 15 cm fixed over the area with tuckers was done to strengthen the posterior wall of the inguinal canal (Fig. 6). Peritoneum was closed over the mesh (Fig. 7). The sac was sent for histopathology which revealed characteristics consistent with hernial sac and no evidence of granuloma or malignancy (Fig. 8). The surgery was well tolerated by the patient who was discharged after 2 days. The patient was encouraged for early ambulation, was prescribed pain killers, and instructed to report to hospital if there is any bleeding or appearance of painful swelling at the site of surgery. She was again seen at follow up 2 weeks later at the surgical outpatient clinic where she had completely recovered from the surgery and was happy. Further follow up 7 months later confirmed no recurrence of the condition.

## 6 Discussion

Anton Nuck, a Dutch anatomist, first described the canal of Nuck in 1691, which is the processes vaginalis within the inguinal canal of females. Failure of obliteration of the distal portion of the canal forms a fluid-containing sac known as the HCN [1]. Among female children, HCN has about 1% incidence [7]. The incidence of HCN in adult females is not entirely clear, probably due to its rarity [7]. Table 1 shows the findings of the literature review of female HCN that were laparoscopically removed. Only three cases were identified, and the table displays and compares our case with the other 3 reported patients.

**Table 1:** Comparison of characteristics of Laparoscopic Hydrocelectomy of the Canal of Nuck in adult Females.

Study	Patient age (year)	Presenting complaint	Size CT (cm)	Size US (cm)	Size MRI (cm)	Laparoscopic Technique	Follow up (months)
Current case 2019 Qatar	36	Painful lump	—	8.4 × 2.6	—	TAPP repair	8
Chihara 2019 Japan	38	Painless lump for 5 months	3 × 3	—	—	TAPP + open posterior approach	19
Matsumoto 2014 Japan	37	Painless lump for 2 years	—	Done but size not mentioned	4.5	TEP repair	NM
Qureshi 2014 India	28	Painful lump for 1 month	4 × 3	—	—	TAPP repair	NM

CT: computerized tomography; US: ultrasound; MRI: magnetic resonance imaging; —: Not done; NM: Not mentioned; TAPP: Transabdominal preperitoneal; TEP: Totally extraperitoneal.

In terms of presentation, HCN may present as painless swelling in the inguinal area and labia majora. Our patient presented with mild painful sausage-shaped swelling extending from the right inguinal region to the right labium major. There were no other findings. Our review further confirmed that the only presenting complaint was painless swelling and no associated symptoms [2,5] and one of them had painful swelling however there were no alarming symptoms [8].

In terms of diagnosis, despite that the ultrasound report of our case that suggested hydrocele, we kept a differential diagnosis of hydrocele vs. oblique inguinal hernia, simply due to the rarity of HCN. We agree that due to its rarity, clinically, HCN can be mistaken for more common groin masses that have similar characteristics such as inguinal hernias, lymphadenopathy, Bartholin's gland cyst, abscesses, and post traumatic hematoma [7,9]. We were able to confirm HCN intraoperatively, in agreement with others that where the majority of the reported cases of HCN were also not conclusively diagnosed until surgery was performed [3]. Our review further confirmed such intraoperative diagnoses of HCN (Table 1) [2,8].

In terms of investigations, imaging studies may aid the pre-operative diagnosis of HCN. HCN displays varied appearances in sonography. In the literature, sonographic appearance of HCN shows thin walled, well defined, anechoic cystic structure that ranges from tubular, sausage, dumbbell or comma-shaped “cyst within a cyst’’ to a multicystic appearance [4]. We are in support, our inguino-labial HCN was a sausage-shaped cystic structure of 8.4 × 2.6 cm. Whilst magnetic resonance imaging can provide more precise images with anatomical relations as it shows better enhancement and diagnosis of encysted structures [10], in our case, ultrasound was undertaken as we did not feel a need for further imaging, given that the laparoscopic surgical approach that we employed is both diagnostic and therapeutic. However, from our review CT scan can be done if suspecting inguinal endometriosis [5]. MRI is also helpful and can guide the surgeon regarding the relations and attachments of the hydrocele with the surrounding tissues [2].

In terms of management, traditionally, treatment of HCN is excision and closure of inguinal ring by open surgery [[11], [12], [13]]. However, some authors suggested that using such open approach, it is not entirely feasible to examine the central aspect of the HCN [5]. Instead, others have suggested initial laparoscopic repair of a suspected hernia, as the direct visualization offered by laparoscope can be used to treat the hernia and also to diagnose other pathologies [14]. Our review (Table 1) suggests that, laparoscopic TAPP excision (transabdominal preperitoneal) and TEP (totally extraperitoneal) approaches have become increasingly popular approaches. Indeed, greater diagnostic potential and excision of encysted hydrocele have been reported using the laparoscopic TAPP approach [14]. In the current case, we used laparoscopic TAPP which was useful in diagnosis and efficient excision of the cyst. We chose TAPP rather than TEP, as TAPP has the added advantage over TEP in that it can exclude any other intraabdominal pathologies that might concurrently exist. To the best of our knowledge, only 3 case reports have been reported golbally [2,5,8].

## 7 Conclusion

This case expands the clinical and intraoperative potential differential diagnosis of adult female groin masses. Surgeons should consider such diagnosis when they observe such swellings intraoperatively. Laparoscopic TAPP approach in such cases is a superior diagnostic and treatment modality.

## Sources of funding

Nothing to declare.

## Ethical approval

Approved by the Medical Research Center, Hamad Medical Corporation reference number (MRC-04-19-119).

## Consent

Written informed consent was obtained from the patient for publication of this case report and accompanying images. A copy of the written consent is available for review by the Editor-in-Chief of this journal on request.

## Author contribution

Fakhar Shahid: study concept, data collection, interpretation, writing the paper.

Walid EL Ansari: data interpretation, writing the paper.

Mohamed Ben-Gashir: pathology, review of the paper.

Abdelrahman Abdelaal: study concept, data interpretation, writing the paper.

## Registration of research studies

1. Name of the registry: NA.
2. Unique Identifying number or registration ID: NA.
3. Hyperlink to the registration: NA.

No required as journal instructions as not first in man study.

## Guarantor

Walid El Ansari: welansari9@gmail.com.

## Provenance and peer review

Not commissioned, externally peer-reviewed.

## Declaration of Competing Interest

Nothing to declare.
